# T cell phenotypes in COVID-19 - a living review

**DOI:** 10.1093/oxfimm/iqaa007

**Published:** 2020-12-29

**Authors:** Stephanie J Hanna, Amy S Codd, Ester Gea-Mallorqui, D Oliver Scourfield, Felix C Richter, Kristin Ladell, Mariana Borsa, Ewoud B Compeer, Owen R Moon, Sarah A E Galloway, Sandra Dimonte, Lorenzo Capitani, Freya R Shepherd, Joseph D Wilson, Lion F K Uhl, The Oxford-Cardiff COVID-19 Literature Consortium, David J Ahern, Hannah Almuttaqi, Dominic S Alonzi, Aljawharah Alrubayyi, Ghada Alsaleh, Valentina M T Bart, Vicky Batchelor, Rebecca Bayliss, Dorothée L Berthold, Jelena S Bezbradica, Tehmina Bharuchq, Helene Borrmann, Mariana Borsa, Rowie Borst, Juliane Brun, Stephanie Burnell, Lorenzo Capitani, Athena Cavounidis, Lucy Chapman, Anne Chauveau, Liliana Cifuentes, Amy Susan Codd, Ewoud Bernardus Compeer, Clarissa Coveney, Amy Cross, Sara Danielli, Luke C Davies, Calliope A Dendrou, Sandra Dimonte, Ruban Rex Peter Durairaj, Lynn B Dustin, Arthur Dyer, Ceri Fielding, Fabian Fischer, Awen Gallimore, Sarah Galloway, Anís Gammage, Ester Gea-Mallorquí, Andrew Godkin, Stephanie J Hanna, Cornelia Heuberger, Sarah Hulin-Curtis, Fadi Issa, Emma Jones, Ruth Jones, Kristin Ladell, Sarah N Lauder, Kate Liddiard, Petros Ligoxygakis, Fangfang Lu, Bruce MacLachlan, Shayda Maleki-Toyserkani, Elizabeth H Mann, Anna M Marzeda, Reginald James Matthews, Julie M Mazet, Anita Milicic, Emma Mitchell, Owen Moon, Van Dien Nguyen, Miriam O'Hanlon, Clara Eléonore Pavillet, Dimitra Peppa, Ana Pires, Eleanor Pring, Max Quastel, Sophie Reed, Jan Rehwinkel, Niamh Richmond, Felix Clemens Richter, Alice J B Robinson, Patrícia R S Rodrigues, Pragati Sabberwal, Arvind Sami, Raphael Sanches Peres, Quentin Sattentau, Barbora Schonfeldova, David Oliver Scourfield, Tharini A Selvakumar, Freya R Shepherd, Cariad Shorten, Anna Katharina Simon, Adrian L Smith, Alicia Teijeira Crespo, Michael Tellier, Emily Thornton, Lion F K Uhl, Erinke van Grinsven, Angus K T Wann, Richard Williams, Joseph D Wilson, Dingxi Zhou, Zihan Zhu, Awen M Gallimore, Anita Milicic

**Affiliations:** 1 Division of Infection and Immunity, School of Medicine, Cardiff University, Heath Park, Cardiff, CF14 4XN, UK; 2 Nuffield Department of Medicine, University of Oxford, Old Road Campus, Roosevelt Drive, Headington, Oxford, OX3 7FZ, UK; 3 Kennedy Institute of Rheumatology, NDORMS, University of Oxford, OX3 FTY, UK; 4 Medical Sciences Division, University of Oxford, Headington, Oxford, OX3 9DU; 5 Nuffield Department of Medicine, The Jenner Institute, University of Oxford, Old Road Campus Research Building, Roosevelt Drive, Oxford, OX3 7DQ, UK

**Keywords:** T cells, COVID-19, phenotypes, antigen-specific, lung, peripheral blood

## Abstract

COVID-19 is characterized by profound lymphopenia in the peripheral blood, and the remaining T cells display altered phenotypes, characterized by a spectrum of activation and exhaustion. However, antigen-specific T cell responses are emerging as a crucial mechanism for both clearance of the virus and as the most likely route to long-lasting immune memory that would protect against re-infection. Therefore, T cell responses are also of considerable interest in vaccine development. Furthermore, persistent alterations in T cell subset composition and function post-infection have important implications for patients’ long-term immune function. In this review, we examine T cell phenotypes, including those of innate T cells, in both peripheral blood and lungs, and consider how key markers of activation and exhaustion correlate with, and may be able to predict, disease severity. We focus on SARS-CoV-2-specific T cells to elucidate markers that may indicate formation of antigen-specific T cell memory. We also examine peripheral T cell phenotypes in recovery and the likelihood of long-lasting immune disruption. Finally, we discuss T cell phenotypes in the lung as important drivers of both virus clearance and tissue damage. As our knowledge of the adaptive immune response to COVID-19 rapidly evolves, it has become clear that while some areas of the T cell response have been investigated in some detail, others, such as the T cell response in children remain largely unexplored. Therefore, this review will also highlight areas where T cell phenotypes require urgent characterisation.

## INTRODUCTION

The T cell arm of the adaptive immune system is vital in the host defence against viral pathogens and in long-lasting immune memory that prevents reinfection. Therefore, an understanding of T cell phenotypes and functions is crucial to enable better treatment of COVID-19, caused by the novel virus SARS-CoV-2. SARS-CoV-2 belongs to the coronavirus family which includes SARS-CoV-1, MERS-CoV and ‘common cold’ coronaviruses (OC43, HKU1, 229E and NL63) [1]. SARS-CoV-1 and MERS-CoV both cause severe disease with high fatality rates. SARS-CoV-1 infection is characterized by lymphopenia and reduced activation of T cells [2], while in MERS-CoV infection lymphopenia is less prevalent, T cells can be directly infected by MERS-CoV and mount a Th17-driven response [3]. In both cases, long-lived memory T cells are generated [4, 5]. The T cell response to SARS-CoV-2 is strikingly different from that which occurs in response to influenza [6, 7]. Direct comparisons between patients with influenza and COVID-19 (including patients from both groups with acute respiratory failure) demonstrate that those with influenza had increased IFN pathway responses and reduced TNF and IL-1β responses compared to patients with COVID-19 [6, 7]. COVID-19 is characterized by lymphopenia [8] and T cell phenotypes are drastically altered (Box 1). Furthermore, it is becoming clear that these phenotypic changes may be important in determining the course of disease [9] (Box 2). A pattern is emerging of heterogeneous T cell phenotypes which display elements of activation and proliferation but also exhaustion and lack of cytokine production. This review examines this heterogeneity and how it correlates with disease severity. SARS-CoV-2-specific T cell phenotypes and T cell phenotypes in the lung and in recovery are also discussed, along with indications of T cell memory formation.

### T cell phenotypes in the peripheral blood

Most studies have examined the phenotype of all T cells in the peripheral blood of COVID-19 patients, however, some studies have used tetramers or *in vitro* activation assays to identify and characterize SARS-CoV-2 specific T cells. The ‘T cell phenotypes in the peripheral blood’ section of this review will examine the gross phenotypic changes seen across T cells as a whole in the peripheral blood (of which the majority will not be specific for SARS-CoV-2), while the ‘SARS-CoV-2 antigen-specific T cells’ section will focus upon SARS-CoV-2 antigen-specific T cells.

#### T cell subsets

In COVID-19, there is a striking loss of T cells, particularly of naïve CD4^+^ T cells [10, 11], but many effector and memory subsets are proportionally increased (although due to lymphopenia this may still represent a reduction in absolute numbers). Changes in specific subsets and correlation with disease severity are summarized in Table 1. It is not clear whether naïve cells are converting to an effector/memory phenotype or lost from the periphery altogether. Most likely, given the substantial reductions in T cell counts, both mechanisms occur.

**Table 1:** Summary of T cell subset perturbation in peripheral blood in COVID-19

Subset	Change in COVID-19 (as percentage of all T cells)	Higher proportions of this subset correlate with:	References
Naïve T cells	Decrease		[10]
Naïve CD4^+^ T cells		More severe disease	[12]
Activated T_fh_	Increased	More severe disease	[13, 14]
Th17	Increased		[14]
Non-conventional (CCR6^+^CCR4^+^ CD161^+^IL-1RI^+^) Th1 (Th1^∗^)	Increased		[14]
Tregs	Increased in some	More severe disease	[12, 14, 15]
Activated CD4 T_CM_-like	Increased		[10]
CD4^+^ T_EMRA_	Increased	Milder disease	[10, 16, 17]
T_CM_		Milder disease	[16]
CD4^+^ memory cells		Milder disease	[12]
Th2		More severe disease	[8, 12]
CD8^+^ T_EMRA_	Increased	Milder disease	[10, 16]
CD8^+^CD27^−^CCR7^+^ T_EM_	Increased		[10]
CD8^+^CD27^+^CCR7^−^ T_EM_	Decreased		[10]
CD4^+^CD8^+^ double‐positive T cells	Increased		[18]
MAIT	Decreased (in periphery)	Milder disease	[19, 20]

#### Proliferation

Numbers of proliferating T cells have been reported to increase in most COVID-19 patients [21, 22], although up to a third of patients have no increase in the percentage of KI-67^+^ cells compared to healthy donors [10]. Many T cells subsets proliferate during COVID-19, including those that are usually quiescent, such as T_CM_ (central memory) and T_EM_ (effector memory) subsets [10] with 10-fold increases in the percentage of blood CD4^+^ and CD8^+^ T_EM_ cells in G1 or S-G2/M cell cycle phases [23]. Furthermore, subsets may be proliferating even though they are decreased in frequency [10]. Proliferation rate is likely influenced by disease stage and severity and enhanced in severe disease [13]. Proliferation of CD4^+^ T cells also correlates with anti-SARS-CoV-2 IgG [23].

#### T cell phenotypic markers and cytokine production: activation and exhaustion

**Figure 1:**
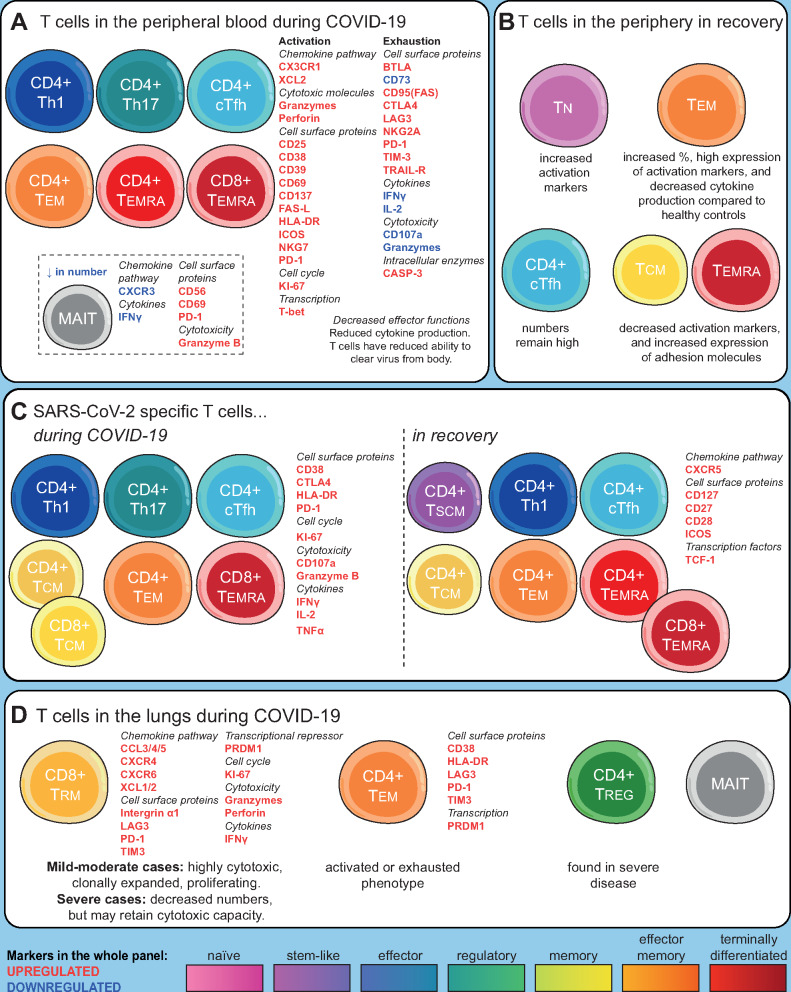
An overview of T cell phenotypes in COVID-19. (**A**) T cell phenotypes in the peripheral blood are characterized by a loss of naïve T cells and an increase in many memory T cell subsets. T cells are activated upon recognition of virus, or in response to cytokines, for example, IL-6 or IFNγ. Activation and antiviral activity are followed by exhaustion and reduction in cytokine production, although the balance between activation and exhaustion correlates with, and may contribute to, disease severity. Decreased effector functions (particularly in CD8^+^ T cells), and reduced cytokine production result in a reduced ability to clear virus from body. (**B**) In recovery, while some phenotypic changes return to normal, others persist (over the timescale currently available to investigators). This includes a persistent elevation of T_fh_ cells, an increased percentage of T_EM_ that over time are replaced by T_CM_ or T_EMRA_, persistent exhaustion of effectors, and increased activation of naïve cells. (**C**) SARS-CoV-2-specific T cells have a range of effector, memory and follicular helper phenotypes and display a range of activation markers and cytokine production, with relatively little evidence of exhaustion. In the recovery phase of COVID-19, these antigen-specific cells convert to long-lived T_CM_ and T_SCM_ phenotypes, with antigen-specific T_fh_ cells also persisting. (**D**) In the lungs, the T cell response is dominated by clonal expansions of CD8^+^T_RM_ (resident memory) cells, although in severe disease, CD4^+^ T_EM_ and Tregs are also found. Exhaustion of CD8^+^ T cells in the lungs appears less pronounced than in the periphery.

CD8^+^ T cells in COVID-19 patients have phenotypes associated with activation, cytotoxicity and cytokine production [22] (Figure 1A). There is an increased frequency of activated HLA-DR^+^ CD38^+^ T cells [10, 13, 23, 24], particularly among memory T cells and CD8^+^ T cells in patients who later progress to severe disease [8, 13, 23]. Over the course of active infection there is a decline in activated CD8^+^CD38^+^HLA‐DR^+^ T cells [18]. Non-naïve CD8^+^ T cells are enriched for expression of CD39, CD27, PD1, ICOS and CD95 (FAS) [10]. CD4^+^ and CD8^+^ T cells in infected patients express high levels of activation markers such as CD25 [25] and CD69 [26] (although whether this increases [27] or decreases [28] over time is unresolved) and effector markers such as NKG7 [22], while overexpression of hypoxia-inducible factor-1 (HIF-1) in T cells suggests an adaptation to hypoxic conditions [23]. CD4^+^ T_CM_ cells have increased expression of activation markers including PD-1 (used as both a marker of activation and exhaustion), CD95 and ICOS [10]. T_EM_ and circulating T follicular helper cells (cT_fh_) show high levels of proliferation and HLA-DR/CD38 co-expression [10]. A good prognosis is indicated by higher levels of CXCR3^+^ CD4^+^ T cells [12] and high expression of CD69 [28] at the time of hospital admission. The most severe disease is associated with decreased frequencies of CD11a^+^ T cells and CD28^+^ CD4^+^ T cells [16] and increased HLA-DR^+^ CD57^+^ T cells [26].

Most, but not all [21], studies have also reported an exhausted [29] or senescent [30] T cell phenotype, particularly in CD8^+^ T cells [29], and link disease severity with decreased polyfunctionality and cytotoxicity [29, 31, 32]. Exhaustion markers increase as disease progresses [31] and high levels of exhaustion markers correlate with a poor prognosis at the time of hospital admission [29]. PD-1 [23, 25, 31], Tim-3 [31, 33], CTLA4 [23, 34], LAG-3 [23], BTLA (B and T lymphocyte attenuator) [14] and the inhibitory receptor NKG2A [32] have been identified as markers of chronic activation, inhibition and an exhaustion in both CD4^+^ and CD8^+^ T cells. CD4^+^ T cells upregulate PD-1 in all subsets except naïve populations [10, 13, 14, 26] and this is more pronounced in severe disease [13]. CD73 is also downregulated [14], again suggesting exhaustion. Overexpression of TRAIL-receptor and CASP3 suggested that CD8^+^ T_EM_ were more prone to apoptosis [23]. There are conflicting reports of TIGIT expression being downregulated [23], or increased [14] in T cells. It has also been suggested that TIGIT’s ligand PVR may directly interact with SARS-CoV-2 [23].

Polyfunctionality in viral infections is often related to a better immune control of the infection [35]. In COVID-19, the loss of cytokine production (IL-2, IFNγ or TNFα) [29] and antiviral activity (CD107a and Granzyme B) [32] in both CD4^+^ and CD8^+^ T cells may be an important contributing factor to disease severity [21, 30]. Elderly (>80 years old) patients in particular had an impaired CD8^+^ T cell cytotoxic response [36]. However, some reports found that T cells in COVID-19 patients, including those with severe disease, expressed substantial amounts of IFNγ [9, 37], IL-17 [9], FASLG, Granzyme A and perforin [23]. In addition, CD8^+^ T_EF_ expressed PTGDR, a mediator of airway inflammation, Granzyme K and XCL2 [37], while T_EMRA_-like populations expressed CX3CR1, T-bet and Granzyme B [10]. Therefore, an alternative possibility is that these functional responses exacerbate COVID-19 disease. Little is known about asymptomatic disease, but one study suggests that these individuals have increased IFNγ, XCL2 and CD69 expression on T cells, and expansion of a Th2-like subset expressing Tumor necrosis factor receptor superfamily, member 19 (TNFRSF19) [38].

A picture emerges of initial T cell activation in response to infection and cytokine-driven bystander activation—indeed, many phenotypic changes correlate with levels of IL-6 or CXCL10 in the serum [23]. T cell activation then progresses to exhaustion, although it is clear that many individual cells simultaneously express markers of both activation and exhaustion [23]. This occurs in the context of viral evasion [39] and impaired innate responses including IFN Type I production [40], which results in reduced virus clearance. It has been proposed that the higher viral titre (due to the reduced viral clearance) leads to further T cell priming [41]. In mild disease, appropriate activation is followed by exhaustion whereas in severe disease excessive activation may be followed by more profound exhaustion.

#### Innate and γδ T cells

Innate and γδ T cells are activated and expanded by non-peptidic antigens and aid elimination of viral and bacterial infections [42]. In COVID-19, circulating and lung innate natural killer T cells (NKT) and mucosal-associated invariant T cells (MAIT cells) exhibit increased expression of both CD69 and PD-1 with reduced secretion of IFN-γ, suggesting that they are both activated and exhausted [28]. MAIT cell CD69^high^, PD-1^high^ and CXCR3^low^ phenotypes associated with poor outcome [19]. In addition, MAIT cells show increased CD56 expression and Granzyme B production [20]. A reduction in the proportion of CD8^+^ MAIT cells [13] and γδ T cells has been observed in the peripheral blood, with Vγ9Vδ2 cells nearly completely absent [23, 43], particularly in patients with severe disease. The remaining γδ T cells are predominantly Vδ1^+^ and highly proliferative, with over 10-fold increases in the proportion of γδ T cells in early cell cycle and G1 [23], while many of the remaining Vγ9Vδ2 cells transition to a memory phenotype over the course of infection [43]. γδ T cells in COVID-19 also express high levels of CD25 [44]. NKT-like cells have increased production of Granzyme B and perforin and their numbers are similar to healthy controls in mild disease, expanded in moderate COVID-19 [45], and decreased in severe disease [17].

#### T cell phenotypes in convalescent COVID-19 patients

After SARS-CoV-2 has been cleared from the body, T cell counts seem to recover to near-normal levels for most patients, although the trajectory of this recovery is associated with the extent of depletion [10, 13, 46, 47]. Abnormalities in T cell phenotypes also persist after the resolution of infection (Figure 1B). During recovery, effector T cells convert to several different memory subsets [16, 17], with a prolonged conversion to T_CM_ [37] and a stem-like memory phenotype [26]. In addition, during early stages of recovery, CD4^+^ T cells express genes associated with positive regulation of cell killing [37] while at later stages they express genes associated with migration and adhesion [37]. Th2-like ICOS^+^ T_fh_ cells are enriched in recovering patients [37] and activated circulating T_fh_ (cT_fh_) frequencies are higher in recovered patients than healthy donors [10], indicating support for B cell antibody production (although there are reports of reduced T_fh_ in recovered patients [46] or at the level of healthy controls [13]). However, there are indications that T cell effector functions may remain impaired in recovered patients. More than a month after hospital discharge, recovered patients had higher levels of CD8^+^ T_EF_ and T_EM_, increased levels of IL-7R on naïve CD8^+^ T cells compared to healthy controls [37], reduced production of IFNγ, IL-4, IL-17 and Granzyme B by both CD4^+^ and CD8^+^ T cells, and increased Treg frequencies compared to uninfected individuals [46]. There are conflicting reports of PD-1 expression in recovered patients being similar to healthy controls [34, 46] or remaining elevated [10], while TIM3 expression on T cells remains high [46]. MAIT cell levels normalized in the convalescent phase [19], while NKT cell numbers remained low 2 months after infection had resolved [46].

It is possible that these T cell perturbations may recover over a longer period than is currently available to assess. However, if persistent, these phenotypes could bring an increased susceptibility to infections and autoimmune diseases.

### SARS-CoV-2 antigen-specific T cells

Most studies have examined the phenotype of all cells in the peripheral blood, however, some studies have used tetramers or *in vitro* activation assays to identify and characterize SARS-CoV-2 specific cells. Antigen-specific CD8^+^ T cells, particularly in the early stages of infection, express CD38, HLA-DR, KI-67 and PD-1 [26] (Figure 1C). They also have effector functions, expressing IFNγ, CD107a, FasL, CCL3, CCL4, Granzyme B [32] and TNF [26], although T cells from patients with severe disease are less likely to be polyfunctional [34]. Expression of many of these, along with clonal expansion, is associated with severe disease [48]. SARS-CoV-2-specific CD4^+^ T cells predominantly had a central memory (CD45RA^−^CCR7^+^) phenotype [27], with spike glycoprotein (S) reactive CD4^+^ T cells displaying an activated/proliferating phenotype (CD38^+^HLA-DR^+^KI-67^+^PD-1^+^) [26]. SARS-CoV-2-specific T cells from recovered patients were able to express IL-2, IFNγ, TNFα [4, 26, 34] and Granzyme B [32].

Intriguingly, T cells with different SARS-CoV-2 epitope specificities have different phenotypes, with spike-specific CD4^+^ T cells skewed towards cT_fh_, whereas membrane (M) protein-specific and nucleocapsid (N) specific CD4^+^ T cells were predominantly Th1/Th17 [26]. Similarly, M/NP-specific CD8^+^ T cells showed wider functionality than T cells targeting the Spike protein [49]. SARS-CoV-2–specific CD8^+^ T cells include large proportions of both central memory (CD45RA^−^CCR7^+^) and terminally differentiated cells (CD45RA^+^CCR7^−^) [27].

In recovered patients, SARS-CoV-2-specific CD4^+^ cells also have a T_CM_/Th1 phenotype with high levels of CXCR5, ICOS and CD127 [50] and higher levels of CTLA4 than patients with active disease [32]. During recovery, CD8^+^ T cells have an early stem cell memory phenotype (CCR7^+^CD127^+^CD45RA^+^TCF-1^+^) [26]. Others have described the SARS-CoV-2-specific CD8^+^ T cells in recovery as atypical CD27^+^CD28^+^ T_EMRA_ cells (terminally differentiated effector memory cells re‐expressing CD45RA) [50], T_CM_ or T_EM_ [49]. Another study found that CD95^+^CD45RA^+^ T_EMRA_, CCR7^+^ CD45RO^+^ CD27^+^ CD28^+^ CD95+ T_CM_ and CD45RO^+^ CD95^+^ T_EM_ SARS-CoV-2 –specific CD8^+^ T cells corresponded to ‘high prevalence’ peptide responses, which were detected in >35% of donors of each allele group [51]. Comparatively, ‘low prevalence’ responses i.e. those occurring in fewer individuals, comprised mainly CCR7^+^CD45RO^+^CD27^+^CD28^+^CD95^+^ T_CM_ and CCR7^+^CD27^+^CD28^+^CD95^+^CD45RA^+^ T_SCM_ (central memory and stem cell memory, respectively) cells [51]. The high prevalence was also more differentiated (CD57^+^ and CD161^+^) compared to the low prevalence (CCR7^+^CD28^+^CCR7^+^) SARS-CoV-2-specific CD8^+^ T cells [51]. Spike-specific cT_fh_ cells were abundant in recovered patients, (indicating support for B cell responses and antibody production), biased towards cT_fh_17 (CCR6^+^CXCR3^−^), and correlated with neutralizing antibody titres [52]. It is suggested that people with asymptomatic COVID-19 may have expansions of SARS-CoV-2-specific CD4^+^, but not CD8^+^ T cells [38]. Reassuringly, functional, antigen-specific T cells were found in people who had recovered from asymptomatic COVID-19 [26]. Another study of recovered patients found broad SARS-CoV-2-specific CD4^+^ and CD8^+^ T cell responses, with strength and breadth of target peptides correlated to disease severity [49]. In addition, T cell responses correlated with antibody titres [49].

Many reports have found that previous common cold coronavirus infections result in the generation of memory T cells that can cross-react with SARS-CoV-2 [4, 26, 48, 53–56]. Furthermore, in a case study of the T cell repertoire of one donor characterized both prior to and in recovery from COVID-19 infection, it was found that pre-existing T_CM_ cells had TCRs that recognized SARS-CoV-2 epitopes. However, after SARS-CoV-2 infection, these SARS-CoV-2 reactive TCRs were now found in the T_EM_ population, with a small fraction in the T_SCM_ [57]. Overall, SARS-CoV-2-specific cells appear to retain a more activated and less exhausted profile [48, 58].

### T cell phenotypes in the lung

In the airways of moderately severe COVID-19 patients, CD8^+^ T cells display an anti-viral phenotype, with high expression of CCL5 and cytotoxic receptors [58] (Figure 1D). In severe COVID-19, CD8^+^ T cells in the airways show increased proliferation but are decreased in number overall [59], implying that peripheral lymphopenia is not solely due to recruitment of T cells to the lungs. T cells from bronchoalveolar lavage (BAL) of patients with mild disease are differentiated and express Granzyme A, Granzyme K, FASLG and CCL5 as well as XCL1, XCL2 [59], whereas T cells from patients with severe disease appear exhausted, lack Th17 and resident memory markers and effector functions [60]. Tregs were increased in the pleural fluid of a COVID-19 patient, compared to the peripheral blood [61]. CD8^+^ T cells expressing CCL3, CCL4, CCL5 and CXCR4, and CD4^+^ T cells expressing CCR4, CD38, HLA-DR and PD-1 were enriched in the pleural fluid while both CD4^+^ and CD8^+^ T cells expressed LAG-3, TIM-3 and PRDM1, indicating exhaustion, and cytokine production was decreased [62]. MAIT cells are enriched in the lungs [19]. These findings highlight the aberrant responses of T cells in the lung, which appear similar to those in the peripheral blood, with over-activation and exhaustion, as a factor in severe disease.

## CONCLUSIONS

Many questions remain regarding the T cell phenotypes in SARS-CoV-2 infection, in particular as stratified by early/late stages and disease severity. Some patients manage to mount an effective anti-viral response, although most of the data show a dysregulated and inefficient response with T cell over-activation followed by exhaustion in both the periphery and the lungs. T cell profile at the point of hospital admission can give useful indication as to the disease prognosis and stratification of patients at this point could enable use of effective immuno-modulating drugs [11]. Evidence of T cell apoptosis is limited, and the causes of lymphopenia remain unknown. The available data are mostly from advanced infection in hospitalized patients and there is a need to characterize the T cell response in the early stages of severe disease, and over the whole time course of mild and asymptomatic infections [63] and in children [64]. In other settings, such as the treatment of multiple sclerosis with alemtuzumab, rapid reduction in lymphocyte numbers is followed by uneven repopulation of T and B cell subsets, leading to a variety of autoimmune diseases due to a lack of T cell regulation of B cell responses [65] and the homeostatic expansion of autoreactive T cells [66]. Therefore, COVID-19 recovery, where lymphocyte repopulation is accompanied by high levels of T cell activation and increased numbers of activated cT_fh_ harbours a heightened risk of autoimmunity. It is already known that a variety of autoimmune conditions can be induced during COVID-19 infection [67], and that rate of onset of type 1 diabetes appears increased following COVID-19 [68]. Future research will undoubtedly focus on characterizing T cell functional deficits and consequences of a persistent phenotypic perturbation following COVID-19.

Box 1: Knowns and unknowns of T cell phenotypes in COVID-19

- In parallel with overall T cell depletion COVID-19, T cell phenotypes including memory and effector subsets and cT_fh_ are expanded and activated. The mechanisms for this, e.g. antigen-specific responses, bystander activation in response to cytokine milieu, are not well explored.
- HLA-DR, CD38, CD69, PD-1 are key markers of T cell activation. Activation is typically followed by exhaustion (PD-1, Tim-3, CTLA4, LAG-3), although the latter may not occur in SARS-CoV-2-specific T cells.
- Severe disease is associated with:
  - Exhaustion of T cells in the periphery.
  - Loss of polyfunctionality and cytotoxicity in both the T cell population generally, and in SARS-CoV-2-specific T cells.
- Activated SARS-CoV-2-specific T cells during recovery convert to phenotypes suggesting long-lived memory, although expression of markers associated with activation and exhaustion persist.
- The current COVID-19 literature lacks sufficient data on early, mild and asymptomatic disease, and disease in children, which could provide greater insight into the underlying mechanisms for T cell depletion, the fate of ‘missing’ T cells and the turning point from activation to exhaustion.
- More data are also needed to determine whether T cell phenotypes are causative, or merely correlate with disease severity.

Box 2: Importance of T cell phenotype in COVID-19

- Perturbation of T cell phenotypes correlate with disease severity and at the point of hospital admission can predict outcome.
- T cell activation may drive tissue damage.
- Phenotypes indicate generation of long-lived immune memory in recovered patients and suggest avenues to assess promising vaccine candidates in addition to antibody generation.
- Phenotypes in recovered patients indicate long-lasting changes that may affect immune response to other pathogens or increase susceptibility to autoimmune diseases.

## FUNDING 

This study was supported by GW4 BioMed MRC Doctoral Training Partnership (to L.C.), European Union's Horizon 2020 under the Marie Sklodowska–Curie grant agreement 893676 (to M.B.); Tenovus Cancer Care ( to S.A.E.G.); Cardiff University (to O.R.M. and A.S.C.); KTRR Prize Studentship (to L.F.K.U.); Immutep (to D.O.S.); Wellcome Trust (to S.D., F.C.R. and A.S.C.).

## AUTHORS’ CONTRIBUTIONS

A.M. and A.M.G. contributed to conceptualization and supervision of the study; D.O.S., F.C.R. and L.F.K.U. were project administration; M.B. contributed to visualization; S.J.H., A.S.C., O.R.M., S.A.E.G., S.D., L.C., F.R.S., J.D.W. and The Oxford-Cardiff COVID-19 Literature Consortium contributed to writing of the original draft; S.J.H., A.M., A.S.C., E.G.-M., K.L., F.C.R., E.B.C. and A.M.G. contributed to writing—review and editing.

## DATA AVAILABILITY

All data are contained within the manuscript. This review was facilitated by weekly releases of the Oxford-Cardiff COVID-19 Literature Consortium journal club—a database of reviewed articles and journals will be made available on request.

## CONFLICT OF INTEREST STATEMENT

None declared.

## APPENDIX 1: THE OXFORD-CARDIFF COVID19 LITERATURE CONSORTIUM—EXTENSIVE AUTHOR LIST

David J. Ahern^1^, Hannah Almuttaqi^1^, Dominic S. Alonzi^2^, Aljawharah Alrubayyi^3^, Ghada Alsaleh^1^, Valentina M. T. Bart^4^, Vicky Batchelor^1^, Rebecca Bayliss^5^, Dorothée L. Berthold^1^, Jelena S. Bezbradica^1^, Tehmina Bharuchq^2^, Helene Borrmann^3^, Mariana Borsa^1^, Rowie Borst^1^, Juliane Brun^2^, Stephanie Burnell^4^, Lorenzo Capitani^4^, Athena Cavounidis^6^, Lucy Chapman^4^, Anne Chauveau^1^, Liliana Cifuentes^1^, Amy Susan Codd^4^, Ewoud Bernardus Compeer^1^, Clarissa Coveney^1^, Amy Cross^7^, Sara Danielli^1^, Luke C. Davies^4^, Calliope A. Dendrou^8^, Sandra Dimonte^4^, Ruban Rex Peter Durairaj^4^, Lynn B. Dustin^1^, Arthur Dyer^9^, Ceri Fielding^4^, Fabian Fischer^1^, Awen Gallimore^4^, Sarah Galloway^4^, Anís Gammage^1^, Ester Gea-Mallorquí^10^, Andrew Godkin^4^, Stephanie J. Hanna^4^, Cornelia Heuberger^1^, Sarah Hulin-Curtis^4^, Fadi Issa^7^, Emma Jones^4^, Ruth Jones^11^, Kristin Ladell^4^, Sarah N. Lauder^4^, Kate Liddiard^5^, Petros Ligoxygakis^12^, Fangfang Lu^13^, Bruce MacLachlan^4^, Shayda Maleki-Toyserkani^4^, Elizabeth H. Mann^1^, Anna M. Marzeda^1^, Reginald James Matthews^14^, Julie M. Mazet^1^, Anita Milicic^15^, Emma Mitchell^4^, Owen Moon^4^, Van Dien Nguyen^4^, Miriam O'Hanlon^1^, Clara Eléonore Pavillet^16^, Dimitra Peppa^3^, Ana Pires^4^, Eleanor Pring^4^, Max Quastel^10^, Sophie Reed^4^, Jan Rehwinkel^17^, Niamh Richmond^1^, Felix Clemens Richter^1^, Alice J. B. Robinson^1^, Patrícia R. S. Rodrigues^4^, Pragati Sabberwal^4^, Arvind Sami^18^, Raphael Sanches Peres^1^, Quentin Sattentau^13^, Barbora Schonfeldova^1^, David Oliver Scourfield^4^, Tharini A. Selvakumar^10^, Freya R. Shepherd^4^, Cariad Shorten^16^, Anna Katharina Simon^1^, Adrian L. Smith^19^, Alicia Teijeira Crespo^5^, Michael Tellier^13^, Emily Thornton^17^, Lion F. K. Uhl^1^, Erinke van Grinsven^1^, Angus K. T. Wann^1^, Richard Williams^1^, Joseph D. Wilson^16^, Dingxi Zhou^1^ and Zihan Zhu^13^

*Affiliations of Consortium members*

^1^Nuffield Department of Orthopaedics, Rheumatology and Musculoskeletal Sciences, Kennedy Institute of Rheumatology, University of Oxford, Oxford, UK, ^2^Department of Biochemistry, Oxford Glycobiology Institute, University of Oxford, Oxford, UK, ^3^Nuffield Department of Clinical Medicine, Nuffield Department of Medicine, University of Oxford, Oxford, United Kingdom, ^4^Division of Infection and Immunity, School of Medicine, Cardiff University, Cardiff, UK, ^5^Division of Cancer and Genetics, School of Medicine, Cardiff University, Cardiff, UK, ^6^Nuffield Department of Medicine, Translational Gastroenterology Unit, University of Oxford, Oxford, UK, ^7^Nuffield Department of Surgical Sciences, University of Oxford, Oxford, UK, ^8^Nuffield Department of Medicine, Wellcome Centre for Human Genetics, University of Oxford, Oxford, UK, ^9^Department of Oncology, University of Oxford, Oxford, UK, ^10^Nuffield Department of Medicine, University of Oxford, Oxford, UK, ^11^Haydn Ellis Building, Dementia Research Institute, Cardiff University, Cardiff, UK, ^12^Department of Biochemistry, University of Oxford, Oxford, UK, ^13^Medical Science Division, Sir William Dunn School of Pathology, University of Oxford, Oxford, UK, ^14^Centre for Medical Education, School of Medicine, Cardiff University, Cardiff, UK, ^15^Nuffield Department of Medicine, The Jenner Institute, University of Oxford, Oxford, UK, ^16^Medical Sciences Division, University of Oxford, Oxford, UK, ^17^Radcliffe Department of Medicine, Medical Research Council Human Immunology Unit, Medical Research Council Weatherall Institute of Molecular Medicine, University of Oxford, Oxford, UK, ^18^Nuffield Department of Orthopaedics, Botnar Research Centre, Rheumatology and Musculoskeletal Sciences, University of Oxford, Oxford, UK, ^19^Department of Zoology, University of Oxford, Oxford, UK
